# Mercury and Other Remedies in the Treatment of Syphilis

**Published:** 1882-05

**Authors:** 


### MERCURY AND OTHER REMEDIES IN THE TREATMENT OF SYPHILIS.

In the New York *Medical Journal and Obstetrical Review* for March, 1882, Dr. George Henry Fox, Clinical Professor of Diseases of the Skin in the College of Physicians and Surgeons, New York, maintains that mercury, while undoubtedly our most valuable remedy in the medicinal treatment of syphilis, is yet an overrated drug, and is not essential to the cure of the disease. It is best administered internally rather than by inunction, by vapor baths, or by hypodermic injection. The amount usually given is unnecessarily large, and its local irritant effects should be avoided. The duration of its use should vary according to the severity of the case : no absolute rule can be laid down. Iodide of potassium, the author thinks, should not be reserved solely for the late period of the disease, for there is no stage in which either iodine or mercury is incapable of doing good. Instead of the so-called " mixed treatment," he prefers to give the two agents separately. Iodide of potassium ought not to be administered continuously for any great length of time. It does its work quickly or not at all, and when unnecessarily continued is sure to do harm. Very large doses should not be used without the very plainest indications. They are not without their value in certain cases, but iodism has doubtless often been mistaken for the manifestations of syphilis. Iron deserves to be ranked with mercury and iodide of potassium, from its effect on the anæmia that invariably accompanies the early stage of syphilis. Cod-liver oil is another remedy of great value, especially where there is a strumous taint.

